# Supplementary figures and images for: Hydroxychloroquine reduces T cells activation recall antigen responses
Source: PLoS One. 2023 Aug 2;18(8):e0287738. doi: 10.1371/journal.pone.0287738 (PMC10395872; doi:10.1371/journal.pone.0287738)

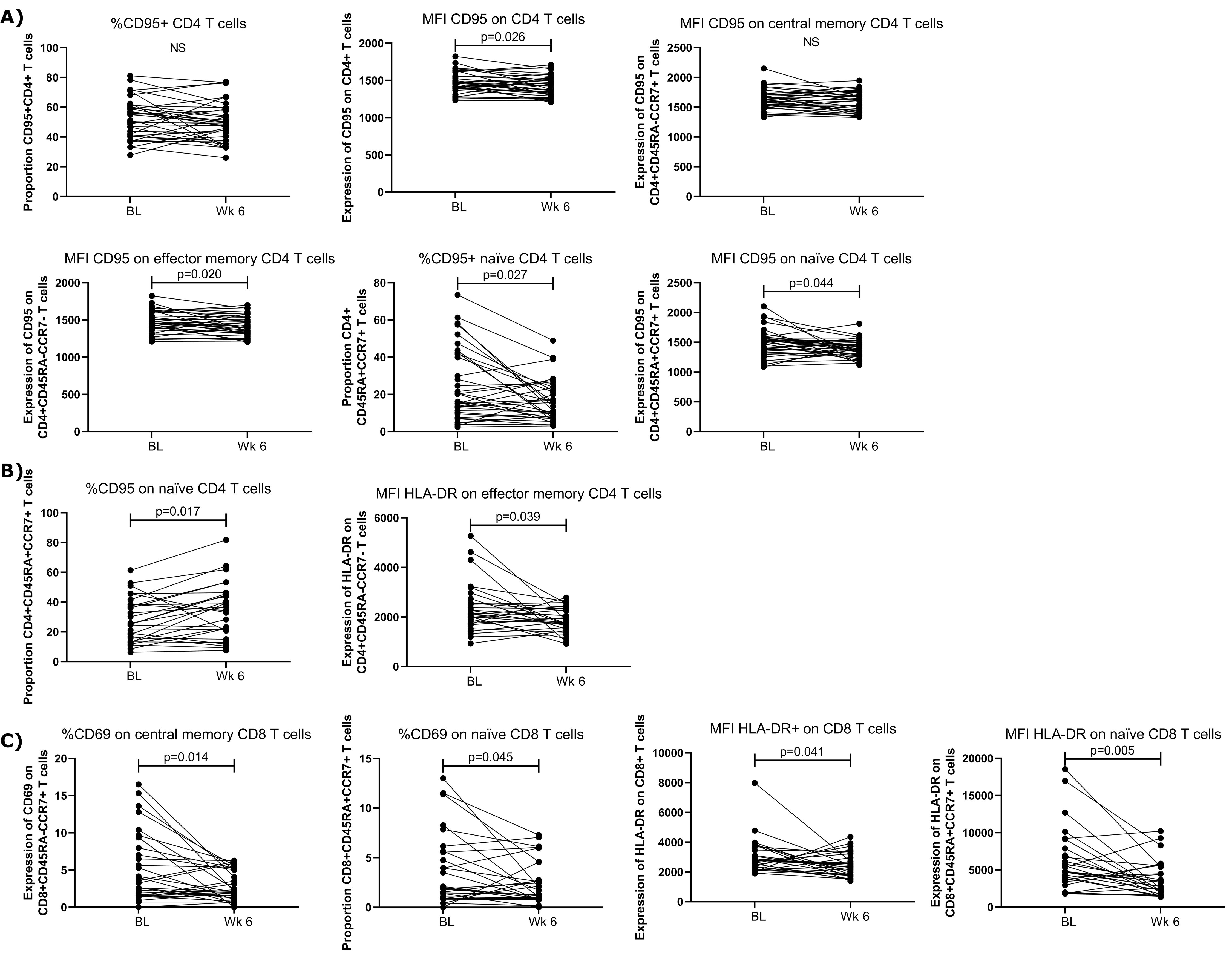

Supplement: S1 Fig — A) Changes in CD4+ T cell markers following 12-hour stimulation. B) Changes in CD4+ T cell markers following 7-day stimulation. C) Changes in CD8+ T cell markers following 7-day stimulation. Data was analyzed using Wilcoxon paired rank test and p values <0.05 were considered significant. (TIF) [file pone.0287738.s001.tif]

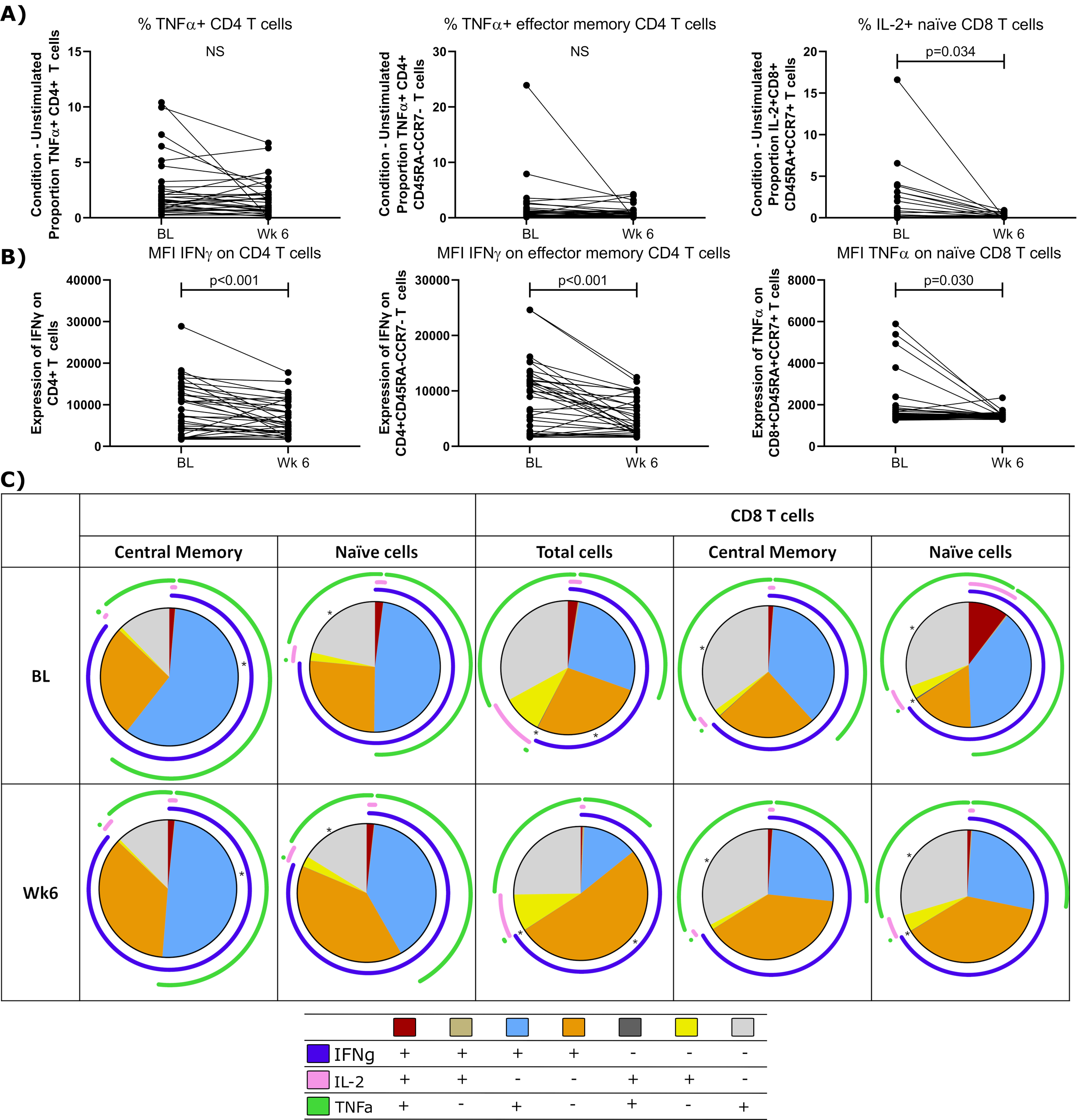

Supplement: S2 Fig — A) Cytokine production determined by subtracting the amount of cytokine detected in the unstimulated condition from each of the stimulated conditions used in this study, B) cytokine expression. C) Co-expression of multiple cytokines, p<0.05 (*), p<0.01 (**), pie colours represent different co-expression options, arc legend colours indicate the three cytokines assessed. Data was analyzed using Wilcoxon paired rank test and p values <0.05 were considered significant. (TIF) [file pone.0287738.s002.tif]

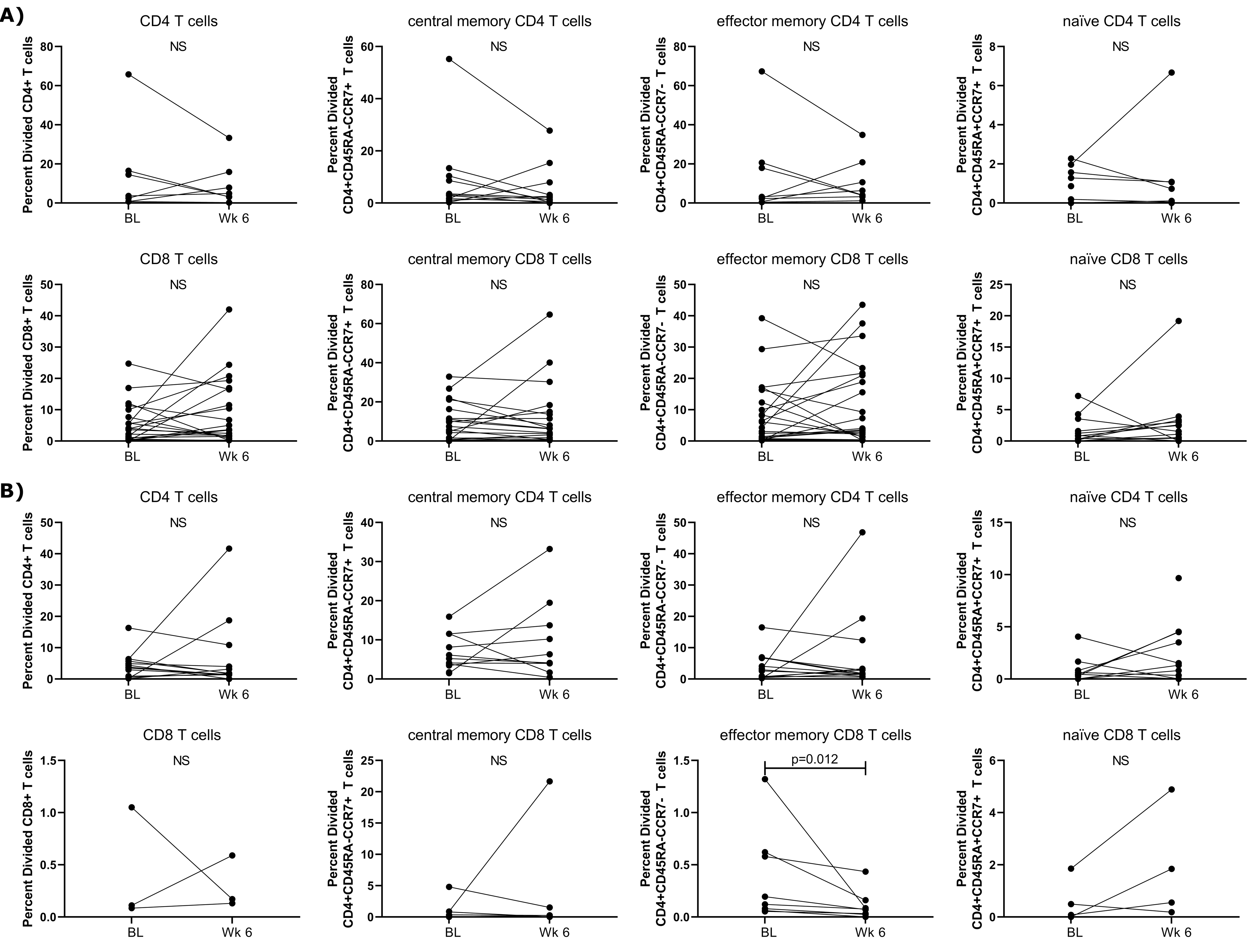

Supplement: S3 Fig — A) Proliferation following stimulation with 2μg/mL CEF (cytomegalovirus, Epstein Barr virus, and influenza virus) peptide pool. B) Proliferation following stimulation with 8μg/mL HPV (human papilloma virus) peptide pool cytokine expression. Data was analyzed using Wilcoxon paired rank test and p values <0.05 were considered significant. (TIF) [file pone.0287738.s003.tif]
