# Supplementary material for: Hydroxychloroquine reduces T cells activation recall antigen responses
Source: PLoS One. 2023 Aug 2;18(8):e0287738. doi: 10.1371/journal.pone.0287738 (PMC10395872; doi:10.1371/journal.pone.0287738)
Supplement: Attachment — Submitted filename: Response to reviewers.pdf [file pone.0287738.s004.pdf]

Rady Faculty of Health Sciences | UM

Dept of Medical Microbiology
and Infectious Diseases

Keith R. Fowke, Ph.D.
Professor and Head,
Medical Microbiology and Infectious Dise[illegible]
Laboratory of Viral Immunology
University of Manitoba
539-745 Bannatyne Avenue
Winnipeg, Manitoba
Canada R3E 0J9
Telephone (204) 789-3818
Fax (204) 789-3926
keith.fowke@umanitoba.ca

Feb 27, 2023

Janin Nouhin, Ph.D.
Academic Editor
PLOS ONE

RE: PONE-D-22-33598
Hydroxychloroquine reduces T cells activation recall antigen responses: Implications for COVID-19 prevention

Dear Dr. Nouhin

Thank you very much for your email of Feb 14, 2023 inviting us to resubmit a revised version of our manuscript: Hydroxychloroquine reduces T cells activation recall antigen responses: Implications for COVID-19 prevention. The suggestions of the reviewers and editorial team have improved the manuscript and we thank them for their comments. We can find below our detailed response to the comments. A revised manuscript with changes tracked has been provided as has an unmarked version. We hope that we have sufficiently addressed the items noted and that the manuscript will be acceptable to the reviewers and editors. We look forward to our manuscript being published in PLOS One.

Best regards,

Keith Fowke

Keith Fowke, PhD
Professor and Head, Dept of Medical Microbiology and Infectious Diseases

Note: Original comments are presented, and our author responses follow in *red italics*.

In response to the comments from the academic editor and journal requirements

We appreciate that the editor indicated that additional experiments are not required.

Additionally, please separate the figure legend from the main text. It would be more appreciated if the figure legends are stated in a separate page.

All figure legends have been moved to 1 page following the reference section.

When submitting your revision, we need you to address these additional requirements.

2. Please ensure that your manuscript meets PLOS ONE's style requirements, including those for file naming. The PLOS ONE style templates can be found at

https://journals.plos.org/plosone/s/file?id=wjVg/PLOSOne_formatting_sample_main_body.pdf and https://journals.plos.org/plosone/s/file?id=ba62/PLOSOne_formatting_sample_title_authors_affiliations.pdf

The submitted file has been updated to match the above style guides.

2. Thank you for stating the following in the Acknowledgments Section of your manuscript:
"Funding support was provided by the Canadian Institutes of Health Research (CIHR) (OCH # 126275) and S5 386-01 from Grand Challenge Canada (JL). "
We note that you have provided additional information within the Acknowledgements Section that is not currently declared in your Funding Statement. Please note that funding information should not appear in the Acknowledgments section or other areas of your manuscript. We will only publish funding information present in the Funding Statement section of the online submission form.
Please remove any funding-related text from the manuscript and let us know how you would like to update your Funding Statement. Currently, your Funding Statement reads as follows:
"KRF (OCH # 126275) from Canadian Institutes of Health Research (https://cihr-irsc.gc.ca/e/193.html). The funders had no role in study design, data collection and analysis, decision to publish, or preparation of the manuscript.
JL (S5 386-01) from Grand Challenges Canada (https://www.grandchallenges.ca/). The funders had no role in study design, data collection and analysis, decision to publish, or preparation of the manuscript."
Please include your amended statements within your cover letter; we will change the online submission form on your behalf.

Funding information has been removed from the acknowledgements section. The current funding statement (as stated above) is correct.

3. We note that you have indicated that data from this study are available upon request. PLOS only allows data to be available upon request if there are legal or ethical restrictions on sharing data publicly. For more information on unacceptable data access restrictions, please see http://journals.plos.org/plosone/s/data-availability#loc-unacceptable-data-access-restrictions.
In your revised cover letter, please address the following prompts:
a) If there are ethical or legal restrictions on sharing a de-identified data set, please explain to them in detail (e.g., data contain potentially sensitive information, data are owned by a third-party organization, etc.) and who has imposed them (e.g., an ethics committee). Please also provide contact information for a data access committee, ethics committee, or other institutional bodies to which data requests may be sent.
b) If there are no restrictions, please upload the minimal anonymized data set necessary to replicate your study findings as either Supporting Information files or to a stable, public repository and provide us with

the relevant URLs, DOIs, or accession numbers. For a list of acceptable repositories, please see http://journals.plos.org/plosone/s/data-availability#loc-recommended-repositories.
We will update your Data Availability statement on your behalf to reflect the information you provide.

We believe in the importance of data sharing. We also recognize the importance of respectful engagement with our community partners. Through the University of Manitoba we will generate a de-identified data set which will be deposited on a publicly accessible data repository platform. This will be communicated with our community partner.

In response to the comments from reviewer 1

This study characterizes T cell responses in healthy individuals administered with HCQ over a period of six weeks. The authors demonstrate decreased T cell activation in samples taken at treatment endpoint compared to baseline. In addition, T cells obtained from these individuals after treatment with HCQ seem to be refractory to short or long stimulation with antigen peptides, as evidenced by their impaired cytokine production. All of these observations are in line with previous literature on the impact of HCQ on T cell responses. Altogether, the authors use the results of this study to advocate for better monitoring of SARS-CoV-2 vaccine responses in individuals on long-term HCQ treatment regimens.

General comments :

There is some literature to suggest that HCQ induces apoptosis of T cells. Therefore, did the authors observe any differences in absolute counts of total T cells, CD4+ or CD8+ T cells or percentages of live/dead cells within the populations gated for T cells, especially at 6 wks post treatment.

Between baseline and Wk6 we did not see differences in the proportion of T cells. As we did not use counting beads during flow cytometry, getting a true absolute count is not possible. However, we did count the cells using trypan blue vitality stain and there was no difference in cell viability between baseline and week 6.

Since CD69 is an early activation marker, did the authors look at its expression on T cells in the 12-hour stimulation conditions? These findings could be included in the manuscript.

CD69 was assessed at the 12-hour stimulation condition however no difference was found between baseline and week6.

In the methodology section, the authors describe an HPLC based method to determine concentrations of HCQ in patient samples. The HCQ concentrations at baseline and Wk. 6 could be included in Table 1. Furthermore, did the concentrations of HCQ correlate with CD4 and CD8 T cells at baseline and Wk. 6?

Plasma drug levels have now been included in Table 1 for plasma. No drug was detected at baseline for any participant therefore no correlations were performed for this time point.

Thank you for the suggestion to perform correlation analyses. We did so on week 6 data, however, none of the major T cell markers showed strong correlations and we felt it did not add much to the paper so did not include that data.

Minor comments :

Materials and methods could be modified to only include methodologies described in the results, e.g. fresh cervical mononuclear cells were not described in the analyses.

The mention of cervical mononuclear cell sample collection and processing has been removed.

Line 250 : Should read “Figure 2C” instead of “Figure 3C”

Thank you for the comment, the figure number has been corrected.

Resolution of the figure images should be improved.

Thank you for the comment, the resolution has been maximized.

In response to the comments from reviewer 2

In this study, the author highlights that hydroxychloroquine (HCQ) treatment used for patients suffering with autoimmune disorders may affect their immune response by reducing the T-cell activation and cytokine production.

Major issues:

1. This study elegantly establishes the relationship between HCQ and immune response, although, no evidence has been provided on HCQ affecting the efficacy of COVID vaccine. Hence, all the text in the manuscript must be corrected to make this point clear.

Thank you for this comment, we agree that we must be clearer in our messaging. To reflect this comment, we have modified the title to remove any reference to COVID-19. We have also made modifications throughout the paper to clarify this issue.

2. Additional validation studies maybe performed to study the levels of T-cell reduction over time

Thank you for this comment, we will be following the editor's instruction to not perform additional studies.

3. Result section needs to modified extensively. The author needs to explain the rationale and the interpretation of their observed results.

In the result section we have added additional information to ensure that there is sufficient rationale described for each section. We have utilized the discussion section for focusing on the interpretation of the results.

4. In the discussion section, the author mentions that their findings help understand "how long-term treatment used by people suffering from immune disorders may impact their immune response". Although, this study has been performed for 6 weeks, so it cannot be called as "long-term" in this manuscript.

We agree with the reviewer's statement and the conclusion has been modified accordingly.

Minor issues:

1. Table. 1 needs to be well structured and formatted.

Reformatted for single space with no blank cells, table legend and title formatted according to the style guide.

2. Title of the manuscript needs to be reframed appropriately.

We agree, the title has been changed. Our new title is "Hydroxychloroquine reduces T cells activation recall antigen responses"
